# Metal–Organic Framework-Based Sensors for Environmental Contaminant Sensing

**DOI:** 10.1007/s40820-018-0218-0

**Published:** 2018-07-13

**Authors:** Xian Fang, Boyang Zong, Shun Mao

**Affiliations:** 1 Biomedical Multidisciplinary Innovation Research Institute, Shanghai East Hospital, State Key Laboratory of Pollution Control and Resource Reuse, College of Environmental Science and Engineering, Tongji University, 1239 Siping Road, Shanghai, 200092 People’s Republic of China; 2 Shanghai Institute of Pollution Control and Ecological Security, Shanghai, 200092 People’s Republic of China

**Keywords:** Metal–organic frameworks, Environmental contaminant, Optical sensor, Electrochemical sensor, Field-effect transistor sensor, Micro- and nanostructure

## Abstract

Increasing demand for timely and accurate environmental pollution monitoring and control requires new sensing techniques with outstanding performance, i.e., high sensitivity, high selectivity, and reliability. Metal–organic frameworks (MOFs), also known as porous coordination polymers, are a fascinating class of highly ordered crystalline coordination polymers formed by the coordination of metal ions/clusters and organic bridging linkers/ligands. Owing to their unique structures and properties, i.e., high surface area, tailorable pore size, high density of active sites, and high catalytic activity, various MOF-based sensing platforms have been reported for environmental contaminant detection including anions, heavy metal ions, organic compounds, and gases. In this review, recent progress in MOF-based environmental sensors is introduced with a focus on optical, electrochemical, and field-effect transistor sensors. The sensors have shown unique and promising performance in water and gas contaminant sensing. Moreover, by incorporation with other functional materials, MOF-based composites can greatly improve the sensor performance. The current limitations and future directions of MOF-based sensors are also discussed.
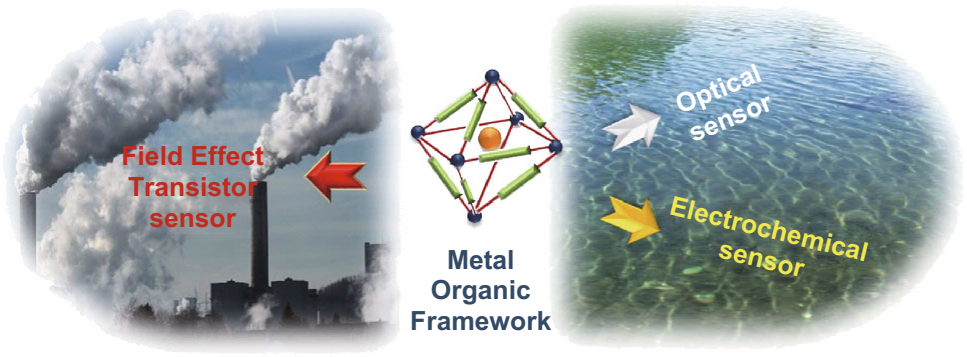

## Highlights

- Representative metal–organic framework (MOF)-based sensing platforms in environmental contaminant detection are introduced.
- The unique structures and properties of MOFs lead to high sensing capabilities in environmental contaminant detection.
- By combining with functional materials, MOF-based composites can improve sensor performance.

## Introduction

In the past decades, with the population boom and industry development, environmental pollution has become a serious problem for the ecosystem and public health. Many types of pollutants, e.g., water and air pollution of heavy metals, organic compounds, and toxic gases, are associated with health risks [1]. Chromatography and its coupled techniques are the most widely used methods in determining environmental contaminants [2, 3]. However, chromatography-based techniques often require expensive equipment, complex pretreatment, and long test times. Thus, new sensing technologies, which posses the advantages of high sensitivity, rapid detection, ease of use, and suitability for in situ, real-time, and continuous monitoring of environmental pollutants, are highly needed.

A sensor is normally composed of a sensing unit and a transduction unit to translate the sensed information into another type of signal, e.g., an electrical or optical signal. The working principle of a sensor is based on its transduction mechanism, which is based on changes in the optical, electrical, photophysical, or mechanical properties of the sensing element in the sensor when it interacts with the analytes [4–6]. In a sensor, important sensing characteristics include sensitivity, selectivity, response time, reusability, long-term stability, and cost [7]. Hence, the selection and design of the sensing material employed in the sensor platform are key points with regard to sensor performance. To date, various micro- and nanomaterials with different characteristics have been employed in environmental monitoring sensors [8], including nanocarbon materials (carbon nanotube and graphene) [9–13], metals and metal oxides [14–16], semiconducting materials [17, 18], quantum dots [19, 20], and polymers [21, 22]. The development of novel sensing materials with excellent properties greatly promotes sensor research and applications.

Metal–organic frameworks (MOFs), also known as porous coordination polymers (PCPs), are a fascinating class of highly ordered crystalline coordination polymers formed by the coordination of metal ions/clusters and organic bridging linkers/ligands [23], which combine the intrinsic merits of the rigid inorganic materials and flexible organic materials. The coordination of metal ions/clusters and ligands results in the formation of extended infinite networks. The concept of MOF was first studied by the Yaghi group in 1995 [24]. They reported a classic MOF structure (MOF-5 based on Zn) with a large specific surface area of 2900 m^2^ g^−1^ and a porosity of 60% in 1999 [25]. Subsequently, many types of MOFs were reported with designed structural, magnetic, electrical, optical, and catalytic properties by choosing appropriate metal ions and organic ligands [26]. Given the wide choices of metal and ligand combinations, MOFs thrive on structural diversity and tunable chemical and physical properties. Owing to their unique structures, MOFs can have an ultrahigh Langmuir surface area (> 10,000 m^2^ g^−1^) [27], which is several times higher than that of activated carbon (1200 m^2^ g^−1^). In addition, the tunable pores and high porosity of MOFs enable their applications in gas storage and separation [28, 29], drug delivery [30, 31], chemical separation [32], sensing [33–35], catalysis [36–38], and bio-imaging [39, 40].

The reversible adsorption, high catalytic activity, tunable chemical functionalization, and diverse structure of MOFs make them ideal sensing elements in chemical sensors [41]. The chemical, physical, and structural changes in an MOF upon adsorption of guest molecules have been utilized in recent years for the detection of environmental contaminants including heavy metals, organic compounds, and toxic gases [42]. This review will introduce recent advances in MOFs-based sensors for the detection of environmental contaminants. Specifically, we will focus on the use of MOF-based materials in optical, electrochemical, and field-effect transistor (FET) sensors. The typical structures and working principles of these sensors will be discussed. Representative examples of these sensors will be introduced, and the critical features and advantages of MOFs that are desired for the sensing performance are highlighted. A summary of current limitations and challenges of MOF-based sensors, and an outlook of the future direction of this emerging sensing material, will also be given.

## MOF-Based Sensors in Aqueous Solution

Water pollutants including heavy metals, anions, organics, antibiotics, and bacteria are harmful to human health and the ecological environment. Therefore, sensitive and reliable sensors for the determination of water contamination are of great significance. MOFs have been widely utilized for chemical detection in an aqueous solution owing to their unique characters for selective capture and determination of analytes. Their porosity and large surface area enable the reversible adsorption and release of target molecules. MOF-based sensors have been used in various sensors relying on luminescent, electrochemical, and colorimetric signals. Although the sensors are based on different sensing mechanisms, the sensing performances of the sensors are promising in water pollutant detection.

### Luminescence Sensors

A considerable amount of work using MOFs as sensing elements for chemical sensors in aqueous solution was based on the luminescence property of MOFs [1, 43]. Luminescence occurs when electrons in excited singlet states return to the ground state via photon emission [44]. This leads to the luminescence phenomenon of the MOF, which is attenuated or quenched upon the absorption of analyte and is known as the “turn-off” mechanism [45]. By contrast, there are also reports in the literature that focus on luminescence enhancement, described as a “turn-on” mechanism [46]. The quantitative and qualitative analysis of analytes can be determined through the luminescence enhancement, quenching, or the movement of the emission wavelength. MOF-based materials are promising as multifunctional luminescent materials since both organic and inorganic components can provide a platform to generate a luminescence signal. The metal–ligand charge transfer-based luminescence within MOFs can bring other dimensional luminescent functionalities; moreover, some guest molecules incorporated in MOFs can emit or induce luminescence [44, 47–52].

#### Inorganic Anion Sensing

Nutrients such as phosphate ions (PO_4_^3−^) in water lead to eutrophication and result in the reduction or elimination of dissolved oxygen, resulting in a negative effect on the water ecosystem [53]. Various luminescent MOF sensors have been employed for the selective detection of nutrient ions. Qian et al. [54] reported on a highly selective photoluminescence quenching-based PO_4_^3−^ sensor with the C_3v_ symmetric cavity of a Tb(III)-based MOF compounds, TbNTA1 (NTA = nitrilotriacetate). This study revealed that inorganic ions including F^−^, Cl^−^, Br^−^, I^−^, NO^3−^, NO^2−^, HCO^3−^, CO_3_^2−^, and SO_4_^2−^ have no impact on the fluorescence intensity of TbNTA1, and only PO_4_^3−^-incorporated TbNTA1 led to a tremendous luminescence quenching effect. The quenching effect can be explained by the matching degree of TbNTA1 with anions. PO_4_^3−^ anions have a tetrahedral shape, and after being incorporated with TbNTA1, the Tb–O bond may weaken the energy that is transferred to Tb^3+^ via nonradioactive relaxation, inducing the luminescence quenching effect. The matching degree of TbNTA1 with PO_4_^3−^ anions is determined by the anion size and pH value. This work was the first report of MOF sensors for highly selective sensing of PO_4_^3−^ anions in aqueous solution.

The Lu group developed an effective fluorescent sensing platform for phosphate detection based on MOF and ZnO quantum dot (QD) conjugates [55]. The MOF material (MOF-5) was used as the substrate, and positively charged ZnO QDs capped by (3-aminopropyl) trimethoxysilane (APTMS–ZnO QDs) were attached to the negatively charged MOFs via amine–Zn interaction and electrostatic interaction. This interaction resulted in the ZnO QD fluorescence quenching owing to the electron transfer process. After introducing phosphate into the QD–MOF system, the presence of phosphate could inhibit the quenching effect and recover the fluorescence of ZnO QDs. The fluorescence intensity depended on the phosphate concentration and was not affected by other interfering species. This fluorescent sensing platform had good sensitivity with a linear working range of 0.5–12 μM and a detection limit of 53 nM. In this study, the sensing platform also shows a satisfactory sensing performance with real water samples. However, such luminescence-functionalized MOF (LMOF) sensors are not reusable.

The first report on reusable MOF-based phosphate sensors was recently presented. In this report, a water-stable 3D-MOF Eu-BTB (based on H_3_BTB = 1,3,5-benzenetribenzoate) was exploited for gleaning an excellent phosphate-selective sensing performance [56]. The luminescence intensity of MOF compounds remained unchanged even after five runs of testing, indicating that interaction between the Eu-BTBn and PO_4_^3−^ is weak and the luminescence recovery may be owing to the removal of PO_4_^3−^.

**Fig. 1:**
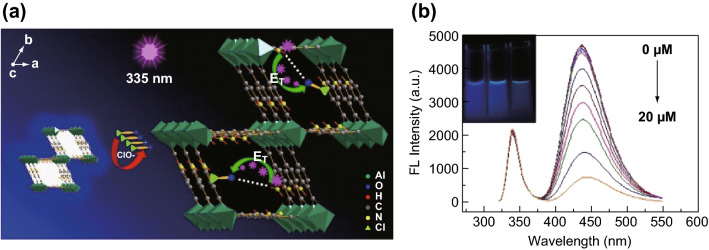
**a** Working principle of NH_2_-MIL-53-based sensor for ClO^−^ sensing. **b** Fluorescence emission spectra of NH_2_-MIL-53(Al)-based sensor toward various concentrations of ClO^−^: 0, 0.05, 0.1, 0.5, 0.8, 1, 2, 3, 5, 7, 10, 15, and 20 μM from top to bottom. Inset shows corresponding photographs of NH_2_-MIL-53(Al) nanoplates in the absence (left) and presence of 10 μM (middle) and 20 μM (right) ClO^−^ under 365 nm UV light. Reprinted with permission from [57]. Copyright (2016) American Chemical Society

It is of great importance to monitor and control the free ClO^−^ ions in drinking water because low-level free ClO^−^ ions cannot kill viruses or pathogenic bacteria effectively, while a higher level may produce many disinfect byproducts (DBPs), which are harmful to human health. Recently, Lu and coworkers proposed a novel fluorescent sensing platform to detect free chlorine based on MOF hybrid materials [57]. The NH_2_-MIL-53(Al) was synthesized by a facile one-step hydrothermal treatment of AlCl_3_·6H_2_O and NH_2_-H_2_BDC in water with urea as a modulator. The as-synthesized Al nanoplates exhibited excellent water solubility and stability. The strong fluorescence of Al nanoplates was significantly suppressed after the addition of free chlorine (Fig. 1a). The sensor platform had a good detection limit of 0.04 μM and a wide detection range from 0.05 to 15 μM (Fig. 1b). The mechanism study suggested that the energy transfer through N–H···O–Cl hydrogen bonding interactions between the amino group and ClO^−^ ions plays a key role in fluorescence suppression. Recovery tests with real tap water and swimming pool water samples showed that the recoveries for real water sample determination were 97–101%.

In addition to the abovementioned inorganic anions, the cyanide ion (CN^−^) is another important contaminant in water, as it is one of the most toxic and lethal pollutants presently occurring in nature. The Ghosh group reported on a selective and sensitive fluorescent senor for the aqueous-phase detection of CN^−^ using an MOF-based system [58]. In this study, a post-synthetic modification of ZIF-90 with the specific recognition sites for CN^−^ was applied. This could selectively sense CN^−^ at 2 μM and fulfilled the permitted contamination limit of 2 mM in drinking water set by the World Health Organization (WHO).

#### Heavy Metal Ion Sensing

Heavy metal ions are one of the nonbiodegradable pollutants in the water environment. Some heavy metals ions including copper (Cu), lead (Pb), mercury (Hg), arsenic (As), chromium (Cr), and cadmium (Cd) are considered to be highly toxic and hazardous to human health even at a trace level. Therefore, many MOF-based sensors have been reported for heavy metal ions detection in water. Lin et al. [59] reported on a highly sensitive and selective fluorescent MOF-based probe for copper ion (Cu^2+^) detection. The branched poly (ethylenimine)-capped CQDs (BPEI-CQDs) with strong fluorescent activity (quantum yield > 40%) and excellent selectivity for sensing Cu^2+^ ions was encapsulated into a zeolitic imidazolate framework (ZIF-8). The obtained BPEI-CQDs/ZIF-8 composites have been used for ultrasensitive and highly selective copper ion sensing. ZIF-8 not only exhibits excellent fluorescent activity and selectivity derived from CQDs but can also accumulate Cu^2+^ owing to the high adsorption property. The accumulation effect of MOFs can amplify the sensing signal. The fluorescent intensity of BPEI-CQDs/ZIF-8 was quenched with the presence of Cu^2+^. This sensing platform can detect Cu^2+^ in a wide concentration range of 2–1000 nM and a lower limit of detection (LOD) of 80 pM. Compared with other fluorescent sensors without the amplifying function of MOF or the introduction of a guest luminophore, this sensing platform has a much lower detection limit of approximately two orders of magnitude. This sensor was also applied in real water sample tests and showed good performance. This study indicated that novel sensing platforms can be designed and applied in heavy metal ion detection by incorporating MOFs with fluorescent nanostructures.

**Fig. 2:**
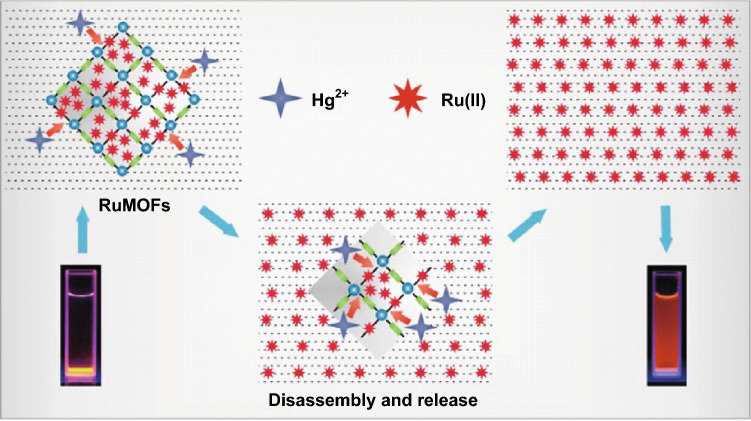
Sensing mechanism of Hg^2+^-responsive disassembly of Ru-MOFs and release of guest material of Ru(bpy)_3_^2+^. Reprinted with permission from [60]. Copyright (2015) American Chemical Society

Mercury ion (Hg^2+^) detection is of special importance given that these substances have high toxicity and present risks to human health. A rapid and selective sensing strategy for detection of Hg^2+^ based on Ru-MOFs was developed by the Chi group [60]. As shown in Fig. 2, the luminescent Ru(bpy)_3_^2+^ was doped in an Ru-MOF framework and encapsulated in the pores of MOF. Before interaction with Hg^2+^, the Ru-MOFs were precipitant in water (yellow powder) and emitted a red color under UV light. However, in the presence of Hg^2+^, the Ru-MOFs were rapidly decomposed by Hg^2+^ ions and released large amounts of luminescent guest materials into the water, i.e., Ru(bpy)_3_^2+^, giving rise to strong fluorescence or electrochemiluminescence signals. As the Ru(bpy)_3_^2+^ was released, the water solution turned yellow and showed red light emission under UV light. The sensor works well when the concentration of Hg^2+^ is in the range of 25 pM to 50 nM with an LOD of 8.2 pM. The LOD was much lower than the United States Environmental Protection Agency (US EPA) mandate of 2 ppb (10 nM) for Hg(II).

Chen et al. [61] designed a turn-on fluorescent sensor based on lanthanide MOF nanoparticles. This sensor can detect Hg^2+^ through an inner-filter effect. Fluorescent Eu-isophthalate MOF nanoparticles were synthesized with an average diameter of 400 nm and exposed to imidazole-4,5-dicarboxylic acid (IDA). The IDA could coordinate with the MOF particle and quench the MOF emission owing to imidazole’s strong absorbance in the MOF excitation region. However, upon Hg^2+^ addition, Hg^2+^ strongly coordinated with IDA and released IDA molecules from the MOF surface, thus restoring the emission. This sensing interaction was both sensitive and selective, with an LOD of 2 nM and negligible responses to Ag^+^, K^+^, Na^+^, Mg^2+^, and Pb^2+^.

#### Organic Compound Sensing

**Fig. 3:**
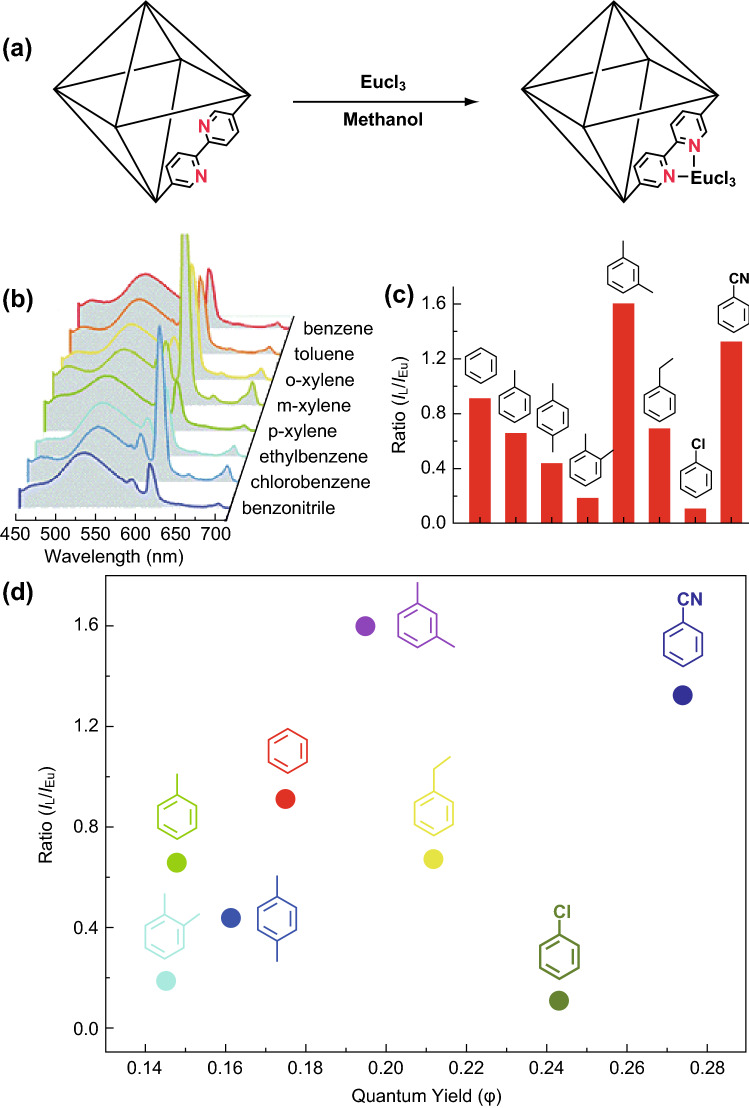
**a** Post-synthetic modification of UiO-bpy with Eu^3+^. **b** Emission profiles and **c** bar diagram depicting relative intensity ratios (*I*_L_/*I*_Eu_), after addition of VOCs. **d** Two-dimensional map displaying relative emission intensities and quantum yields of various VOC encapsulated phases. Reprinted with permission from [63]. Copyright (2016) Royal Society of Chemistry

Among water pollutants, volatile organic compounds (VOCs) such as benzene and its derivative aromatic compounds are substantively toxic pollutants that can cause severe environmental problems and pose threats to the ecosystem [62]. Recently, a fluorescent MOF was established by incorporation of Eu^3+^ cations into a nanocrystalline MOF Zr_6_(u^3^–O)_4_(OH)_4_(bpy)_12_ (recognized as bpy-UiO, bpy = 2,2-bipyridine-5,5-dicarboxylic acid), which could detect aromatic VOCs with high performance [63]. As shown in Fig. 3, the sensor works with an unprecedented dual-readout orthogonal identification scheme. In this sensor, the MOF and VOCs binding event could be recognized with two-dimensional (2D) readouts that combined the intensity ratio of the ligand-based emission to the Eu^3+^ emission (*I*_L_/*I*_Eu_) and luminescence quantum yield. Since the fluorescence characteristics of the MOF nanocomposite rely heavily on the VOCs properties, the retrievable identities of VOCs can be succinctly decoded into an analogous 2D readout (Fig. 3d).

Morsali et al. [64] explored novel sensing probes for the detection of nitroaromatic compounds based on TMU-31 and TMU-32 MOFs. These two MOFs were mixed with nitro-substituted compounds for sensing. In the fluorescence emission spectra, the quenching efficiency of TMU-31 and TMU-32 for nitroaromatic compounds is shown as follows: 1,3-dinitrobenzene (1,3-DNB) > 2,4-dinitrotoluene (2,4-DNT) > nitrobenzene (NB) > nitromethane (NM) > 2,4,6-trinitrotoluene (TNT). This study revealed that the urea groups inside the pore cavity of MOFs work as binding sites for nitroanalytes through N–H···O hydrogen bonds and *π*–*π* stacking interactions. In addition, it was found that the urea group’s orientation inside the pore cavity of MOFs and the supramolecular interactions between the interpenetrated networks are important to nitro-substituted compound sensing.

Zhang et al. reported on an electrochemically synthesized IRMOF-3, which was synthesized by an electrochemical strategy at room temperature for the first time. The electrochemical system consisted of a zinc plate as the anode, a copper plate as the cathode, tetrabutylammonium bromide (TATB) as supporting electrolyte, and DMF–ethanol as solvent. The system exhibited high fluorescent detection properties for 2,4,6-trinitrophenol (TNP) with a detection limit up to 0.1 ppm [65].

Antibiotic tetracycline (TC) is one of main organic contaminates in water and is difficult to degrade in water. The Cuan group developed a dual-functional platform for the detection and removal of TC with a highly stable luminescent zirconium-based MOF (PCN-128Y) [66]. The detection was based on the efficient luminescence quenching of PCN-128Y toward TC. Theoretical and experimental studies revealed that the luminescence quenching can be attributed to a combined effect of the strong absorption of TC at the excitation wavelength and the photo-induced electron transfer process from the ligand of PCN-128Y to TC. The strong metal–ligand bonding between Zr_6_ nodes and TC through solvent-assisted ligand incorporation was suggested to mainly account for the high adsorption capability of PCN-128Y toward TC in water. The preconcentration of TC within the pores of PCN-128Y induced by the adsorption process significantly enhanced the efficiency of TC sensing.

### Electrochemical Sensors

An electrochemical sensor works based on the redox reactions of the analytes in an electrochemical system. The electrochemical measurement is normally carried out using a three-electrode system consisting of a working electrode, a counter electrode, and a reference electrode. The amount of analytes involved in the reaction can be determined by measuring the current, electric potential, or other electrical signals [67, 68]. MOFs show potential as electrochemical sensing surface modifiers because of their high surface area and pore volume, good absorbability, and high catalytic activity [69, 70]. With the rapid development of synthesis methods, stable MOFs with high electrical conductivity have been successfully designed and synthesized [71–73]. However, most MOFs still have poor electrical conductivity and relatively low stability in aqueous solution; this is a result of the reversible nature of the coordination bonds. In addition, MOFs usually have a micron size, resulting in limited adhesion affinity between MOFs and the electrode surface. These disadvantages of MOFs limit their applications in electrochemical sensors. The key to achieving efficient electrochemical signals is to prepare MOFs with high redox activity and electrical conductivity while preserving their unique pore structure. One of the commonly applied methods to resolve these problems is combining MOFs with other functional materials that have high electrical conductivity [33, 74, 75].

#### Ion Sensing

**Fig. 4:**
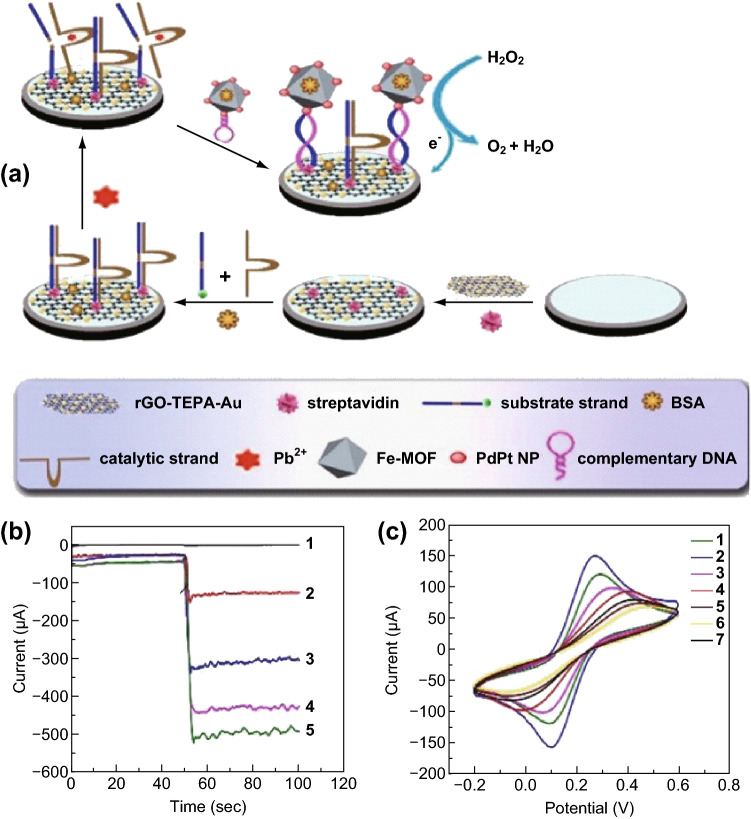
**a** Schematic of sensing strategy. **b** Amperometric *i*–*t* curves of different nanomaterials: (1) Fe-MOFs, (2) Fe-MOFs/AuNPs, (3) Fe-MOFs/PtNPs, (4) Fe-MOFs/PdNPs, and (5) Fe-MOFs/PdPt NPs. **c** CV characterization of electrodes at various stages of modification: (1) bare GCE, (2) rGO-TEPA-Au/GCE, (3) streptavidin/rGO-TEPA-Au/GCE, (4) substrate strand/streptavidin/rGO-TEPA-Au/GCE, (5) BSA/substrate strand/streptavidin/rGO-TEPA-Au/GCE, (6) catalytic strand/BSA/substrate strand/streptavidin/rGO-TEPA-Au/GCE, and (7) Pb^2+^/catalytic strand/BSA/substrate strand/streptavidin/rGO-TEPA-Au/GCE. Reprinted with permission from [76]. Copyright (2018) Elsevier

Lead ion (Pb^2+^) is one of the most toxic and commonly found heavy metal ions in aquatic ecosystems. The monitoring of Pb^2+^, especially trace amounts of Pb^2+^ in the water environment, is important to the public health. Recently, the He group designed a detection strategy to detect Pb^2+^ using Pd–Pt alloy-modified Fe-MOFs (Fe-MOFs/PdPt NPs) with hairpin DNA immobilized on the surface as a signal tag [76]. As shown in Fig. 4, a streptavidin-modified reduced graphene oxide-tetraethylene pentamine-gold nanoparticle (rGO-TEPA-Au) composite serves as the sensor platform for DNAzyme immobilization. In the presence of Pb^2+^, the substrate strand of the DNAzyme is catalytically cleaved, resulting in the detachment of the catalytic strand from the sensor. The newly generated single-strand DNA on the sensor can hybridize with the hairpin DNA. Thus, the hybridized material Fe-MOFs/PdPt NPs is attached to the electrode surface. Fe-MOFs exhibit highly peroxidase activity, and PbPt NPs can enhance the catalysis performance by reducing the H_2_O_2_. Based on the sensing strategy, the amount of Pb^2+^ can be detected by measuring the H_2_O_2_ reduction current. This sensor has a linear working range from 0.005 to 1000 nM and an LOD of 2 pM for Pb^2+^ sensing.

Another electrochemical sensor based on MOFs for Pb^2+^ sensing was designed by Guo et al. [77]. Flake-like MOF material NH_2_-MIL-53(Cr) was prepared using a reflux method and was modified on the surface of a glassy carbon electrode (GCE). This sensor showed excellent electronic responses for Pb^2+^. Under optimal conditions, the oxidation current of Pb^2+^ linearly increased as the concentration increased in the range of 0.4–80 μM with an LOD of 30.5 nM. The NH_2_-MIL-53(Cr)-modified electrode also showed excellent selectivity and stability for Pb^2+^ determination.

Nitrite ion (NO_2_^−^) is regarded as an important contaminant in water. The Mobin group designed a hybrid MOF/rGO electrode for the electrocatalytic oxidative determination of nitrite [78]. In this study, Cu-MOFs were stacked with rGO by a simple ultrasonication method. The GCE modified with Cu-MOF/rGO composites exhibited better electrocatalytic performance for nitrite oxidation (LOD of 0.033 mM) than those of an MOF electrode or bare electrode. The improved sensing performance was owing to the increased conductivity of MOF with rGO. Additionally, this sensor showed good selectivity toward nitrite in the presence of common salts such as CH_3_COONa, KCl, MgSO_4_, CaCl_2_, NaClO_4_, and KNO_3_. This sensor was also tested with NO_2_^−^ spiked pond water with recoveries of 100–120%.

#### Organic Compound Sensing

**Fig. 5:**
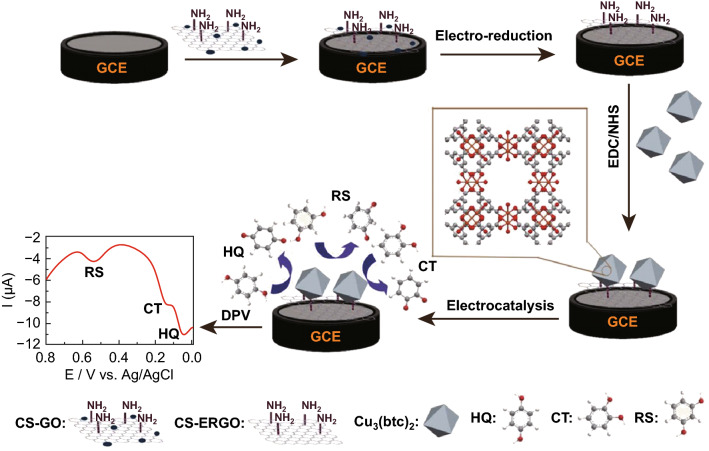
Schematic and detection strategy of electrochemical sensor based on MOF Cu_3_(BTC)_2_ and CS/rGO for DBIs detection. Reprinted with permission from [80]. Copyright (2016) American Chemical Society

Catechol (CT), resorcinol (RS), and hydroquinone (HQ) are three typical dihydroxybenzene isomers (DBIs) of phenolic compounds, which usually coexist as environmental pollutants [79]. An MOF-based electrochemical sensing platform for the simultaneous detection of these DBIs was developed [80]. As shown in Fig. 5, chitosan (CS) was coated on the electrode surface, along with the doping of GO. Through a simple electroreduction method, the GO in the CS/GO composite was electrochemically reduced to rGO, which had a high electrical conductivity and was utilized as the supporting carrier for the grafting of electroactive MOF Cu_3_(BTC)_2_. The good film-forming ability and the covalent binding of GO and CS endowed the sensing surface (Cu_3_(BTC)_2_/ERGO/CS) with a high stability. Electrochemical experiments showed that the reduction peaks of RS, CT, and HQ can be well separated from each other, indicating good selectivity. Meanwhile, the high conductivity of the CS/rGO matrix greatly enhanced the current response, leading to good LODs of 0.44, 0.41, and 0.33 μM for HQ, CT, and RS, respectively. The accurate determination of DBIs in real water samples was also realized by the MOF sensor, which broadened the applications of MOFs in organic compound sensing.

The Wang group designed another electrochemical sensing platform for DBI detection based on MOFs. In this work, magnetic Ni@graphene composites with a core–shell structure (C-SNi@G) were synthesized through the thermal annealing of Ni-BTC MOF [81]. Cyclic voltammetry (CV) measurements showed that only one oxidation peak was found on the bare electrode. This was owing to the overlap of oxidation peaks of HQ and CT, indicating that the HQ and CT could not be differentiated by the bare electrode. However, two distinct oxidation peaks were observed (HQ at 0.085 V and CT at 0.190 V vs. Ag/AgCl, respectively) when the C-SNi@G/MGCE was employed. In addition to the selectivity improvement, the magnetic fabrication of this sensing platform was binder-free and easy to control. The Liu group [82] also reported on a highly sensitive electrochemical sensor for the simultaneous determination of HQ and CT in water based on copper-centered MOF–graphene composites [Cu-MOF-GN, Cu-MOF: Cu_3_(BTC)_2_]. Under optimized conditions, the Cu-MOF-GN electrode showed excellent electrocatalytic activity and high selectivity toward HQ and CT. The detection LODs of HQ and CT were 0.59 and 0.33 μM, respectively. The electrode was also applied in spiked tap water with recoveries from 99.0 to 102.9%.

2,4-dichlorophenol (2,4-DCP) is one of the chlorinated phenol contaminants in water and can accumulate in the human body through the food chain. It is harmful to human health even at a very low concentration [83]. For the sensitive detection of 2,4-DCP, Dong et al. [84] fabricated a simple and rapid electrochemical sensor employing 1,3,5-benzenetricarboxylic acid copper (Cu_3_(BTC)_2_) as the sensing material. The fabricated sensors not only exhibited high selectivity toward 2,4-DCP compared with the interferences, but also showed a wide linear sensing range from 0.04 to 1.0 μM and an LOD of 9 nM. The employment of Cu_3_(BTC)_2_ presents many advantages such as large specific surface area, high adsorption capacity, and good electron transfer efficiency, which enhance the performance of the electrochemical sensor. Furthermore, the sensor was successfully applied for the determination of 2,4-DCP in reservoir raw water samples with satisfactory results.

### Other Sensors in Aqueous Solution

Colorimetric sensors are commonly used in water contaminant sensing based on the chromogenic reaction of colored compounds. The components and amounts of target compounds can be determined by measuring the color change of the solution [85, 86]. The Gu group reported on chromophoric Ru complex-doped MOFs (RuUiO-67) and explored their performance as sensing probes for the colorimetric detection of Hg^2+^ [87]. It was found that thiocyanate-bearing dyes in Ru(H_2_bpydc)(bpy)(NCS)_2_ (H_2_L) complex could specifically interact with Hg^2+^ owing to the strong affinity between Hg^2+^ and the thiocyanate groups in the dyes. Thus, RuUiO-67 served as the recognition site and signal indicator. Upon the addition of Hg^2+^, a concomitant red-to-yellow color change of the probe suspension was recognized, and the detection limit was determined to be as low as 0.5 μM for Hg^2+^.

**Fig. 6:**
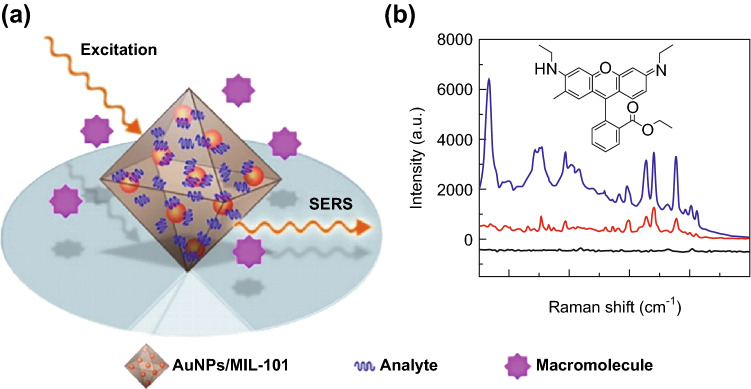
**a** Schematic of MOF-based SERS platform and **b** SERS spectra of rhodamine 6G on Au NP/MIL-101 substrate (blue lines), Au colloids substrate (red line), and MIL-101 (black line). Reprinted with permission from [89]. Copyright (2014) American Chemical Society. (Color figure online)

Surface-enhanced Raman scattering (SERS) is a promising spectroscopic technique for biological and chemical sensing because of its unique advantages such as high sensitivity with the potential of single molecule detection and highly informative spectra characteristics [88]. The signal of SERS strongly depends on the distance between the analytes and the metal nanostructure. For a quantitative analysis of organic pollutant p-phenylenediamine in environmental water, the Li group [89] utilized an Au NP-embedded MOF structure for SERS detection. In this composite, Au NPs were grown and encapsulated within the host matrix of MIL-101 by a solution impregnation strategy. The Au NP/MIL-101 composites combined the localized surface plasmon resonance properties of Au NP and the high adsorption capability of MOF, making them highly sensitive SERS substrates by the effective preconcentration of analytes in close proximity to the electromagnetic fields at the SERS-active metal surface (Fig. 6a). The SERS substrate was sensitive and robust to several different target analytes (Fig. 6b). The substrate also showed high stability and reproducibility owing to the protective shell of the MOF. The practical application potential of the SERS substrate was evaluated by a quantitative analysis of organic pollutant p-phenylenediamine in environmental water and tumor marker alpha-fetoprotein in human serum. The sensor showed good linearity between 1 and 100 ng mL^−1^ for p-phenylenediamine (recoveries: 80.5–114.7%) and 1–130 ng mL^−1^ for alpha-fetoprotein.

## MOF-Based Gas Sensors

MOFs have large intrinsic porosities with > 90% free volumes, adjustable internal surface areas [90–93], and a high degree of crystallinity, which enable the adsorption of guest molecules through strong host–guest interactions [94, 95]. The sustainable pores within MOFs provide a natural habitat for guest molecules, thus increasing the chances for guest–host interactions and the sensing sensitivity [95]. While the sensitivity depends partly on the method of signal transduction, it mainly depends on the strength of analyte binding to the MOF. Thus, stronger binding leads to higher responses [96]. Owing to their unique physical and chemical properties, MOF-based materials show significant promise as sensing materials not only in water contaminant detection but also for gases.

**Fig. 7:**
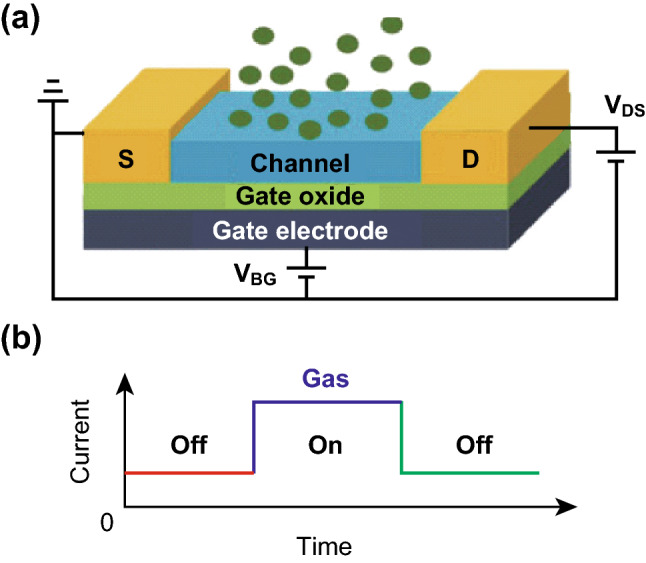
**a** Schematic illustration of FET sensor with source and drain electrodes, channel material, gate oxide, and gate electrode. **b** Sensor current change after gas adsorption and desorption. Reprinted with permission from [97]. Copyright (2017) Royal Society of Chemistry

An FET gas sensor is composed of source and drain electrodes, channel material, gate oxide, and gate electrode [97] (Fig. 7a). The channel material is the key to monitoring the conductance change in the sensor by the physically adsorbed target gases. Therefore, intrinsic properties such as the work function, carrier mobility, and band gap of the channel material determine the sensing performance. The conductance in the channel materials can increase or decrease (depending on the type of the semiconductor and reducing or oxidizing gas) during the interaction between the channel material and gas (Fig. 7b). The adsorbed gas molecules can change the local carrier concentration in the channel material, which leads to the conductance change [98]. FET sensors have been widely used in gas detection as they need no chemical agents and can respond to low-concentration gases instantaneously. Recently, MOF-based materials were applied to FET sensors as the sensing channel or combined with other gas-sensitive materials to improve the sensing capability.

For gas sensing applications, the large capacity and highly selective gas adsorption properties of MOFs are utilized to improve the sensor performance [99–101]. Composite structures that take advantage of the sensitivity of semiconductors and the selectivity of MOFs are desirable [73, 102]. Dinca and coworkers reported on conductive MOF-based FET sensors for ammonia sensing and achieved good sensing performance [109]. He et al. demonstrated noble-metal@MOF composites with highly selective sensing properties to volatile organic compounds, in which the sensor could detect 0.25 ppm formaldehyde at room temperature [111]. To develop highly sensitive and selective FET gas sensors, many studies have focused on developing hybrid nanostructures constructed with MOFs and noble metals or transition metal oxides such as Pd–ZnO/ZnCo_2_O_4_ [103], Au@ZnO@ZIF-8 [104], and Au@MOF-5 (Zn_4_O(BDC)_3_) [105].

**Fig. 8:**
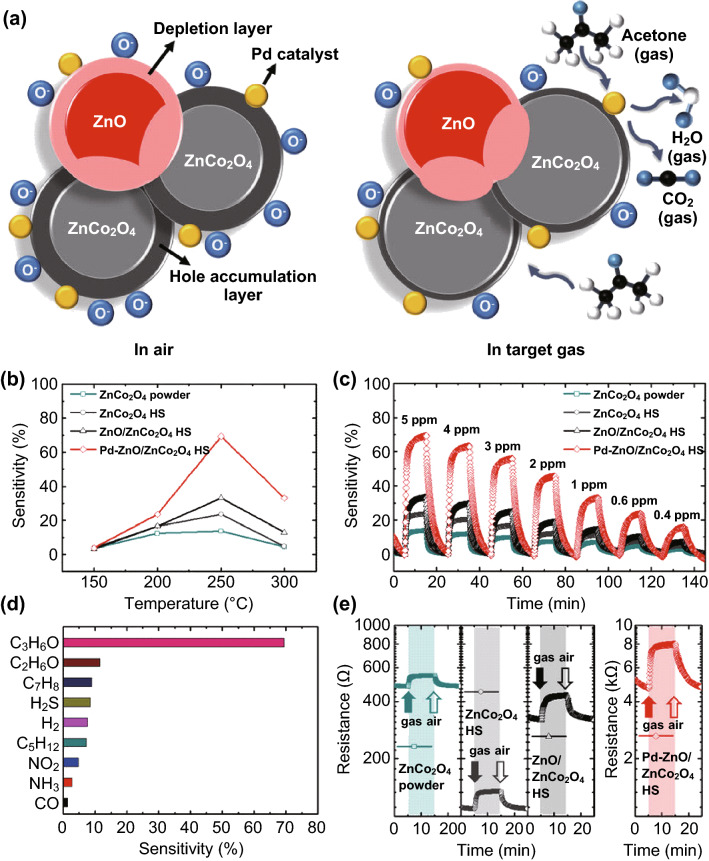
**a** Schematic of acetone sensing mechanism with Pd–ZnO/ZnCo_2_O_4_ hollow spheres. **b** Temperature-dependent acetone sensing characteristics to 5 ppm in temperature range of 150–300 °C. **c** Dynamic acetone sensing responses in concentration range of 0.4–5 ppm at 250 °C of ZnCo_2_O_4_ powders, ZnCo_2_O_4_ hollow spheres, ZnO/ZnCo_2_O_4_ hollow spheres, and Pd–ZnO/ZnCo_2_O_4_ hollow spheres. **d** Selective acetone detection characteristics of Pd–ZnO/ZnCo_2_O_4_ hollow spheres. **e** Dynamic resistance transition properties of samples toward 5 ppm acetone at 250 °C. Reprinted with permission from [103]. Copyright (2017) Macmillan Publishers Limited

Using Pd–ZnO/ZnCo_2_O_4_ hollow spheres, Koo et al. [103] reported a highly sensitive acetone sensor (as shown in Fig. 8a). The ZnCo_2_O_4_ is a p-type semiconductor. Oxygen molecules in the air adsorb on its surface and deprive its electron, followed by creating a hole accumulation on its surface through reactions between chemisorbed oxygen species (O^2−^, O^−^, and O_2_^−^) and a reducing gas such as acetone. As a result, ZnCo_2_O_4_ exhibited high response to acetone (sensitivity = 14% to 5 ppm at 250 °C). The outstanding sensing performance of the sensors is owing to several reasons. At first, the Pd–ZnO/ZnCo_2_O_4_ hollow spheres with high surface area provide many binding sites for acetone, which raises the reaction efficiency. Second, n-type ZnO induces a p–n junction in p-type ZnCo_2_O_4_, followed by recombination between the electron in ZnO and the hole in ZnCo_2_O_4_, thus reducing the hole concentration in ZnCo_2_O_4_. Moreover, the sensor resistance increases because an electronic sensitizer such as Pd has good catalytic properties and can decrease the activation energy [19, 106]. The additional electrons recombine with holes in Pd–ZnO/ZnCo_2_O_4_, and the hole accumulation layer is significantly decreased. Therefore, the acetone sensing response is dramatically increased by the Pd catalyst.

**Fig. 9:**
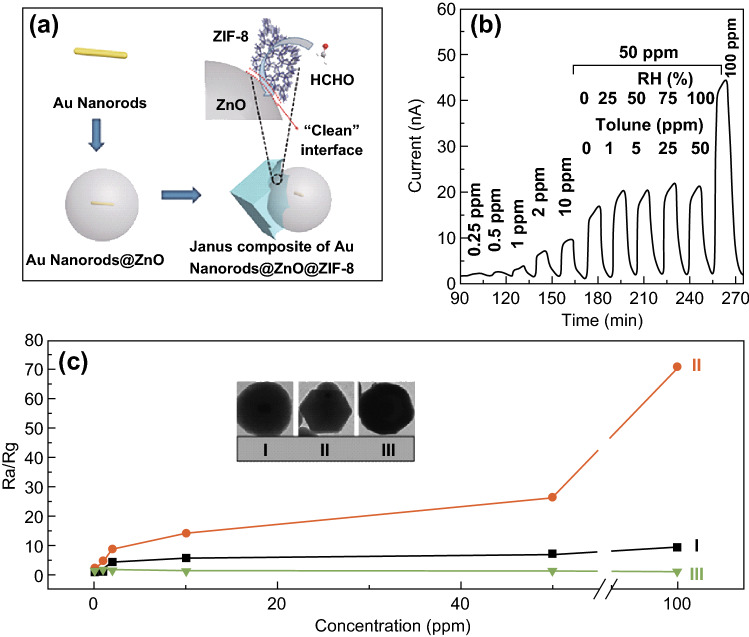
**a** Schematic illustration of anisotropic synthesis of Au@ZnO@ZIF-8. **b** Dynamic response of Au@ZnO@ZIF-8 to HCHO with concentrations from 0.25 to 100 ppm. **c** Plots of sensor responses to HCHO concentration of three samples: (I) pristine Au@ZnO, (II) synthetic Au@ZnO@ZIF-8, and (III) completed Au@ZnO@ZIF-8. Inset shows TEM images of samples. Reprinted with permission from [104]. Copyright (2017) Springer

Another example of an MOF gas sensor was demonstrated by Wang [104], who synthesized a novel composite structure of Au@ZnO@ZIF-8 to simultaneously detect and remove VOCs with photo-induced gas sensing (Fig. 9). Three moieties within the dual-functional nanomaterials were synthesized via an anisotropic growth method. The synergistic effects in sensing and removing were achieved by the plasmonic Au nanorods for plasmonic resonance to enhance the photocatalysis of ZnO, a semiconductor ZnO for high conductivity [106–108], and ZIF-8 for improving the gas adsorption capability.

**Fig. 10:**
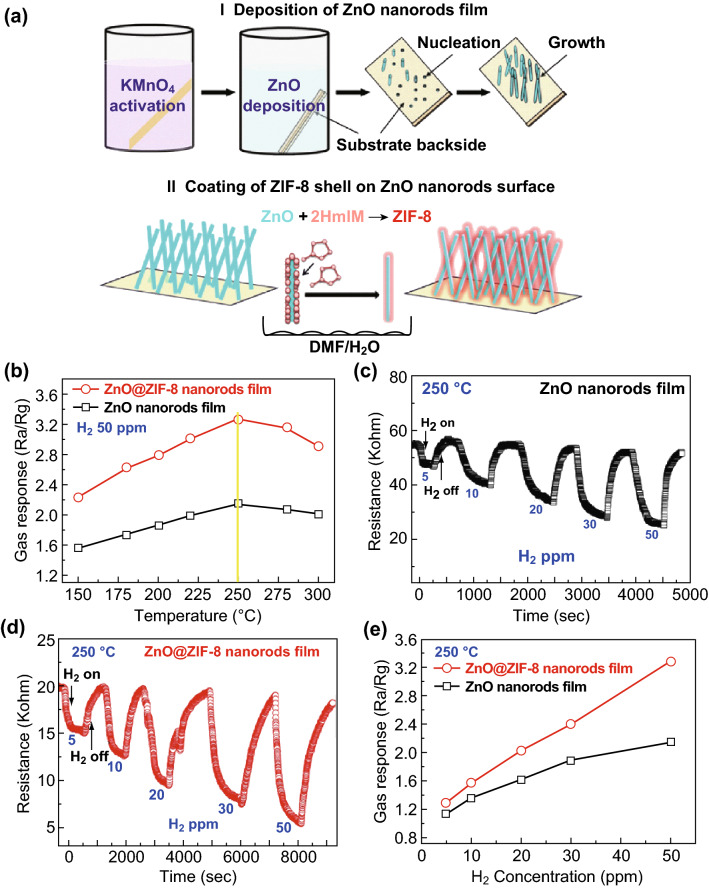
**a** Preparation of ZnO@ZIF-8 core–shell nanorod films. **b** Temperature-dependent response curves of ZnO nanorod and ZnO@ZIF-8 sensors to 50 ppm H_2_. **c**, **d** Dynamic response curves of two sensors to different H_2_ concentrations at 250 °C. **e** Concentration-dependent H_2_ response curves of two sensors at 250 °C. Reprinted with permission from [110]. Copyright (2017) John Wiley & Sons, Inc.

As for the practical applications of gas sensors, MOF-based films have attracted considerable attention owing to their relatively large exposure area to gas and their high stability in the target gas flow [109]. In a recent study, a ZnO@ZIF-8 core–shell nanorod film (thickness of 100 nm) was synthesized to detect H_2_ over CO_2_ through a facile solution deposition process [110]. The ZnO nanorod film was grown on a KMnO_4_-activated glass substrate to form a nanorod structure and ensure direct contact between the film and substrate. The ZIF-8 material had huge cavities of 11.6 Å diameter as well as short pore apertures of 3.4 Å, which exhibited effective separation of H_2_ (2.9 Å) from CO (3.7 Å) owing to the molecular sieving effect [110, 111]. Thus, thin ZnO nanorod film is favorable for H_2_ diffusion owing to its open structure, while ZIF-8 enhances the selectivity of H_2_ sensing over other gases. This sensor can detect low concentrations of H_2_ of 5–50 ppm at 200 °C (Fig. 10).

MOF sensors based on other sensing mechanisms have also been demonstrated for gas detection. The Salama group fabricated a chemical capacitive sensor for the detection of sulfur dioxide (SO_2_) at room temperature [112]. The sensing layer was fabricated with indium MOF (MFM-300), which was deposited on a functionalized capacitive interdigitated electrode. The fabricated sensor exhibited high sensitivity to SO_2_ at concentrations down to 75 ppb and a detection limit of 5 ppb. This remarkable detection is owing to the associated changes in film permittivity upon the adsorption of SO_2_ molecules. The MFM-300 sensor also showed desirable detection selectivity toward SO_2_ in the presence of CH_4_, CO_2_, NO_2_, and H_2_.

Dou and coworkers demonstrated a luminescent MOF film sensor [MIL-100(In) ⊃ Tb^3+^]. This sensor works by quenching the luminescence of the MOF film through bimolecular collisions with O_2_, which possesses high sensitivity and fast response to O_2_ sensing [113]. In another study, Zhang and coworkers introduced Eu^3+^ ions to the free -COOH sites of MIL-124 ligand channels to detect low concentrations of NH_3_ in ambient air, and achieved good sensing performance [114]. The Eddaoudi group utilized the thin film of rare-earth metal (RE)-based MOFs as a sensing platform for the detection of hydrogen sulfide (H_2_S) at room temperature [115]. The RE-MOF offers a distinctive H_2_S detection of concentrations down to 100 ppb with a limit of detection of 5.4 ppb.

To summarize, MOF-based gas sensors have been demonstrated in various gas detection approaches with good sensing performance in terms of fast response and recovery, high sensitivity, high selectivity, and simple test procedures.

## Conclusion and Outlook

Table 1 summarizes MOF-based sensors relying on optical, electrochemical, and FET signals for environmental contaminant sensing. MOFs have been demonstrated as sensing materials for heavy metal, anion, organic compound, and gas detection owing to their unique structure and properties such as large surface area and tunable porosity, reversible adsorption, high catalytic ability, and tunable chemical functionalization. As discussed, MOF-based materials have shown outstanding sensor performance, which can be further improved by combining with other functional materials. For instance, lanthanide-based MOFs (Ln-MOFs) and fluorophore-modified MOFs are promising sensing materials in luminescent sensors as the guest molecules incorporated in MOF can emit or induce luminescence. The combination with high conductive materials (e.g., carbon nanomaterials and noble metals) endows MOF with better stability and electroconductivity when used in electrochemical sensors. In addition, the functional groups incorporated with MOFs can specifically recognize the target analytes and enhance the sensing selectivity.

**Table 1:** MOF-based sensors for water and gas contaminant detection

Sensing material	Method	Target contaminant	LOD	Environmental sample test	References
Eu(III)@UMOFs	luminescent	Hg^2+^, Ag^+^ and S^2−^			[116]
UiO-66-NH_2_	luminescent	PO_4_^3−^	1.25 μM		[117]
APTMS–ZnO QDs@MOF-5	luminescent	PO_4_^3−^	53 nM		[55]
UiO-66-NH_2_	luminescent	Hg^2+^	17.6 nM		[118]
NH_2_-MIL-53(Al)	luminescent	ClO^−^	0.04 μM	tap and swimming pool water	[57]
CDs@Eu-DPA MOFs	luminescent	Cu^2+^	26.3 nM	real water	[119]
Eu–Zn (1·NO_3_^−^) Tb–Zn (2·NO_3_^−^)	luminescent	I^−^	0.001 ppM		[120]
Eu–UiO–66(Zr)–(COOH)_2_	luminescent	Cd^2+^	0.06 μM	environmental water	[121]
Zn_3_(TDPAT)–(H_2_O)_3_	luminescent	nitrobenzene	50 ppM		[122]
H_2_O ⊂ CuI-MOF	luminescent	volatile organic compounds	1 ppm		[123]
Zn_4_O(BDC)_3_	electrochemical	Pb^2+^	4.9 nM	real water	[124]
Cu-MOF/rGO	electrochemical	NO^2−^	33 nM	pond water	[78]
UiO-66-NH_2_	electrochemical	NO^2−^	0.01 μM		[125]
Cu_3_(BTC)_2_	electrochemical	2,4-dichlorophenol	9 nM	reservoir raw water	[84]
Me_2_NH_2_@MOF-1	electrochemical	Cu^2+^	1 pM	river water	[126]
Cu-MOF-199/SWCTs	electrochemical	Hydroquinone and catechol	0.08 and 1 μM	river water	[127]
RuUiO-67	colorimetric	Hg^2+^	0.5 μM		[87]
Tb_1.7_Eu_0.3_(BDC)_3_·(H_2_O)_4_	colorimetric	Cd^2+^	0.25 mM	lead-polluted water samples	[128]
Pd–ZnO/ZnCo_2_O_4_	FET	acetone	0.4–5 ppm		[103]
Cu_3_(HITP)_2_	FET	ammonia	0.5–10 ppm		[73]
ZnO@ZIF-8	FET	formaldehyde	10–200 ppm		[129]
ZIF-67	FET	formaldehyde	5–500 ppm		[111]

Although MOF-based materials have shown a lot of promise, future work is still needed to improve the sensor performance in terms of sensitivity, selectivity, stability, and reusability. First, the function of MOF in each sensing platform should be better understood to fully utilize the advantages of MOF and to assist with sensor design and performance optimization. Second, it is worthwhile to further broaden the MOF category with new useful properties such as plasmonic, electrical, and thermochromic properties. This will provide more opportunities in sensor design and integration. Third, MOF’s pore structure is critical to many sensing application, and new synthesis or surface functionalization methods are needed to better tune the MOF structure and enhance its sensing activity and stability. In electrochemical and FET sensors, the sensing materials not only need to have high activity but also acceptable conductivity. Therefore, the conductivity of MOF should be increased by either developing conductive MOF materials or combining MOF with other conductive substrates, e.g., conductive nanocarbons. Moreover, the stability of MOF-based sensing materials needs to be improved, especially for sensors that work under an acid condition. Finally, most of the sensor demonstrations were conducted using laboratory-prepared samples. Therefore, the sensor performance in a complex environmental media, such as real water, needs to be evaluated and the sensor selectivity and reliability should be improved as they are the two major critical requirements for practical use of the sensors.
